# Benign Paroxysmal Positional Vertigo in Irradiated Nasopharyngeal Carcinoma Survivors

**DOI:** 10.1155/2013/698575

**Published:** 2013-10-24

**Authors:** Shaoyan Feng, Yunping Fan, Liqing Guo, Zibin Liang, Jiaoping Mi

**Affiliations:** ^1^Department of Otolaryngology, The Fifth Affiliated Hospital of Sun Yat-Sen University, No. 52 Meihua East Road, Xiangzhou District, Zhuhai 519020, China; ^2^Department of Radiation Oncology, The Fifth Affiliated Hospital of Sun Yat-Sen University, No. 52 Meihua East Road, Xiangzhou District, Zhuhai 519020, China

## Abstract

*Purpose*. It has been assumed that postirradiated nasopharyngeal carcinoma (NPC) patients are prone to benign paroxysmal positional vertigo (BPPV). The purpose of this study was to better understand this clinical entity. *Materials and Methods*. From September 2003 to June 2011, we conducted a retrospective study of 11 irradiated NPC patients with BPPV in our institute. During the same period, 11 irradiated NPC patients without BPPV were randomly selected and enrolled as the control group. All medical records of these patients were evaluated. *Results*. The risk of BPPV rises significantly when the patient undergoes radiotherapy (RT) twice and the threshold radiation dose is >120 Gy (*P* = 0.027). The occurrence of postirradiated BPPV was significantly related to incidences of otitis media and sensorineural hearing loss (SNHL) (*P* = 0.011 and 0.009, resp.). All the patients responded well to repositioning maneuvers. *Conclusion*. A second course of RT, postirradiated otitis media, or SNHL is associated with the potential risk of radiation-induced BPPV. Repositioning maneuvers were safe and effective for relief of this disease.

## 1. Introduction

 Nasopharyngeal carcinoma (NPC) is a tumor arising from the epithelial cells that cover the surface and line of the nasopharynx. Radiotherapy (RT) is by far the most primary treatment of NPC. Due to complex anatomy, exposure of adjacent and nontarget organs during irradiation of the nasopharynx areas is frequently unavoidable. Ondrey et al. [1] studied the radiation exposure of otologic structures in head and neck cancer and implied that the temporal bone received a significant dose of radiation. Consequently, with the increasing number of irradiated NPC survivors, more and more patients are experiencing otologic and neurotologic complications, such as otitis media with effusion, sensorineural hearing loss (SNHL), dizziness/vertigo, and oscillopsia [2–5]. However, benign paroxysmal positional vertigo (BPPV) in irradiated NPC patients remains scarcely reported [6].

 BPPV is a syndrome characterized by short-lived episodes of vertigo (a sensation of instability, often with a sensation of rotation) in association with rapid changes in head position. There are a number of aetiologies associated with BPPV, such as vertebrobasilar ischaemia, labyrinthitis, ear surgery, head trauma, and vestibular neuritis [7]. Because the radiation portals for NPC consist of the bilateral temporal bones, whether it is radiation damage to the inner ear that leads to BPPV in irradiated NPC patients remains unexplored. The purpose of this study is to investigate the possible causes of postirradiation BPPV and to better understand this clinical entity via retrospectively reviewing postirradiated NPC survivors with BPPV. We think this paper presents our experience with radiotherapy-related BPPV in patients with NPC and may be helpful in the evaluation and treatment of similar patients.

## 2. Materials and Methods

 Our hospital is a major referral center for NPC patients who need radiotherapy, and 1205 patients have been treated and followed up at the Department of Radiation Oncology since 2002. When the otologic complications occurred in these NPC patients, they are referred to our Department of Otolaryngology. During the study period, there were 11 irradiated NPC patients who suffered from BPPV in this population. We conducted a retrospective study of these patients in our institute. During the same period, 11 irradiated NPC patients without BPPV who were matched for tumor stage, age, and gender with the study group were randomly selected and enrolled as the control group. All patients were staged according to the American Joint Committee on Cancer Staging Manual [8]. This study was approved by the Institutional Review Board of the Fifth Affiliated Hospital of Sun Yat-sen University. 

 Efforts were made to collect data on the radiotherapy time, dose, fractionation, and portal arrangements for all the patients. 22 patients were irradiated by using two opposing lateral fields and an anterior field. They received external beam radiotherapy with 6 MV linear accelerators at our hospital, using a 2 Gy daily fraction, and the prescription dose was 70–78 Gy/35–39 f. Stage I and II patients received RT alone. In those with Stage III or IV, two courses of concurrent chemotherapy were administered (cisplatin 60 mg/m^2^ and 5-fluorouracil 600 mg/m^2^) at weeks 1 and 5. They were diagnosed as having local tumor relapse if recurrent nasopharyngeal carcinoma was confirmed by biopsy. Patients who were diagnosed with local tumor relapse all received second course RT in this study. 

 All these treated patients had undergone otoscopic examination of the eardrums, local checkup of nose and nasopharynx field, neurologic examination, blood examination, audiometry, electronystagmography (ENG), and magnetic resonance imaging (MRI). The diagnosis of chronic otitis media (COM) was made when patients had a perforated ear drum and purulent ear discharge. Otitis media with effusion (OME) were diagnosed by clinical symptoms, otoscopic examination, and audiometry. SNHL was defined as bone conduction threshold deterioration of more than 30 dB in the pure tone average (PTA). The diagnosis criteria of BPPV were (1) history of repeated brief episodes of vertigo with changes in head position; (2) vertigo provoked by the Dix-Hallpike or the supine roll test; (3) detectable nystagmus by naked-eye examination or ENG, during and after the provoking maneuvers. Patients with any clinical, laboratory, or imaging findings suggesting pathology of the central nervous system were excluded in our study. 

 Patients with positive Dix-Hallpike maneuver were diagnosed with posterior canal BPPV and treated by Epley's maneuver or Semont's maneuver [9], whereas patients with positive supine roll test were diagnosed with horizontal canal BPPV and treated by the Lempert maneuver [10] or Gufoni's maneuver [11]. In case of failure or incomplete remission of symptoms, the same maneuver was repeated every 3 to 5 days, until a maximum of 3 repetitions. Assessment of the success of the treatment included both patients' relief from vertigo and negative Dix-Hallpike or supine roll test for vertigo and nystagmus. All the patients were followed up for at least 6 months. 

 Because the total number of the patients was less than 40, clinical characteristics were compared between the two groups by using Fisher's exact test. A value of *P* < 0.05 (two-sided) was considered statistically significant. Statistical analysis was performed using the SPSS 17.0 statistical software.

## 3. Results

 Clinical characteristics of these 11 patients with postirradiated BPPV are shown in Table 1. The latency period between the final RT session and the occurrence of BPPV ranged from 3 to 7 years (mean: 5.2 years). Of the 11 patients with BPPV, 4 patients (4/11, 36.4%) had undergone RT once and the nasopharyngeal accumulated RT dose was 70–78 Gy. The rest 7 patients (7/11, 63.6%) had undergone RT twice and the RT dose was 120–130 Gy. Marked neck fibrosis was present in 3 patients (3/11, 27.3%). Otoscopic examination revealed that 4 patients (5 ears) experienced chronic otitis media and 5 patients (7 ears) had otitis media with effusion, for a total of 9 patients (9/11, 81.8%) with middle ear complications. Audiometry revealed that sensorineural hearing loss occurred after RT in 8 patients (14 ears, 72.7%). All patients denied using ototoxic otic drops and previous ear trauma. At the time of this analysis, 3 of the 11 patients died and 8 patients survived. The cause of death for the 3 patients was bleeding in 1 and distant metastases in 2.

Comparison of the clinical characteristics of 11 postirradiated NPC patients with BPPV and 11 postirradiated NPC patients without BPPV is shown in Table 2. The radiation dose in the 11 patients with BPPV was 70–78 Gy in 4 patients and 120–128 Gy in 7 patients, indicating that the risk of BPPV rises significantly when the patient undergoes RT twice and the threshold radiation dose is >120 Gy (*P* = 0.027). The occurrence of postirradiated BPPV was significantly related to incidences of otitis media (COM or OME) and sensorineural hearing loss (*P* = 0.011 and 0.009, resp.). Although the incidences of neck fibrosis were higher in patients with BPPV, there were no significant differences between the two groups (*P* = 1.000).

 In 8 patients (72.7%), the unilateral posterior canal was involved, as evidenced by vertigo and torsional upbeating nystagmus provoked unilaterally during the Dix-Hallpike test. In 3 patients (27.3%), vertigo and geotropic/apogeotropic horizontal nystagmus were provoked during the supine roll test, implying involvement of the horizontal canal. None presented with anterior canal. All the patients responded well to repositioning maneuvers, with resolution of positional vertigo and nystagmus (Table 3). No patients were lost to followup.

## 4. Discussion

Although it has been reported that postirradiated NPC patients are prone to vertigo [12], we found that there are few articles related to the clinical manifestation or prognosis of benign paroxysmal positional vertigo. No studies have identified the risk factors of this disease in these patients either. In our study, 11 postirradiated NPC patients with BPPV had been studied. 

 Radiation damage to the inner ear and radiation-induced vascular insufficiency have been proposed as the aetiology of postirradiated BPPV. More than 50% of the total radiation dose is delivered to the inner ear during head-and-neck cancer RT [13]. For example, conventional fractionated RT (2 Gy per day with a total dose of 60 Gy) to the inner ear of guinea pigs induced atrophy and degeneration of stria vascularis, a reduced number of capillaries, degeneration of endotheliocyte in vessels, outer hair cell, and supporting cell of Corti's organ, and degeneration of vestibular hair cell and otolith seperated from the utricular macula [14]. Gabriele et al. [15] also reported that 44% of patients developed vestibular disorders when the inner ear received 28–51.2 Gy of radiation. In the current study, high radiation dose and a second course of radiation can increase the risk of postirradiated BPPV, further supporting that radiation damage to the inner ear correlates with BPPV.

 In the present study, an association between postirradiated otitis media and postirradiated BPPV was observed. Postirradiated otitis media are considered as a complication of radiotherapy, arising from impairment of the middle ear mucosa and dysfunction of the Eustachian tube after irradiation [16]. Postirradiated SNHL, another complication of radiotherapy, is due to the irradiation damage to Corti's organ and acoustic nerve and was found as a risk factor of BPPV in our study. All these radiotherapy-induced ear injuries seem to be a predicting factor for development of postirradiated BPPV. It is unlikely that hyperemia, edema, and desquamation of mucosa of the middle ear or Corti's organ after irradiation can cause postirradiated BPPV, but development of postirradiated otitis media or SNHL can just be another manifestation of radiation damage and indicates individual sensitivity to radiation. The presence of radiotherapy-induced ear complication would, thus, be evidence of radiation complication and points to an increased risk of damage to the vestibular apparatus and development of postirradiated BPPV.

 Postirradiated BPPV, just like common BPPV, is caused by debris in the semicircular canal of the ear that continues to move after the head has stopped moving. Patients with this disorder complain of episodic vertigo of brief duration, provoked by head movements and accompanied by a characteristic paroxysmal positional nystagmus. The nystagmus is typically torsional-vertical or horizontal, depending on the semicircular canal involved, and is characterized by findings such as latency, crescendo and decrescendo, transience, reversibility, and fatigability [17]. Diagnosis is easily obtained using the Dix-Hallpike and the supine roll tests. Application of repositioning maneuvers, which intends to move particles from the semicircular canal into the vestibule, results in successful treatment of the disease in most patients rather than medication. Some postirradiated NPC survivors had marked neck fibrosis and it was difficult for them to perform the Lempert maneuver, which involved rolling the patient 360 degrees in a series of steps to effect particle repositioning. Gufoni's maneuver is easy to perform in these patients. This maneuver is more tolerable than the Lempert maneuver and showed a good outcome. This study showed that repositioning maneuvers could provide an effective treatment result in patients with postirradiated BPPV. However, considering the low incidence of postirradiated BPPV, the effectiveness of repositioning maneuvers for the treatment of this disease needs to be further studied in a larger group of patients.

 Our results suggest that therapeutic benefits of a second course of RT are associated with the potential risk of radiation-induced BPPV. Postirradiated NPC patients with otitis media or SNHL are prone to have postirradiated BPPV. In this study, the clinical manifestations of postirradiated BPPV in NPC patients varied, and repositioning maneuvers were safe and effective for relief of the vertiginous symptoms.

## Figures and Tables

**Table 1:** Clinical characteristics of 11 irradiated NPC patients with BPPV.

Patient	Gender	Stage	Age at RT (yrs)	RT dose (Gy)	Neck fibrosis	Latency (yrs)	Otitis media	Outcome
1	F	II	53	122	N	3	COM	Alive
2	M	II	45	74	Y	5	OME	Died
3	M	III	32	124	N	7	OME	Alive
4	M	IV	44	120	N	4	COM	Died
5	F	I	60	70	N	6	N	Alive
6	M	I	57	126	Y	6	OME	Alive
7	M	II	43	72	N	7	COM	Alive
8	F	II	65	120	N	3	N	Alive
9	M	IV	51	128	Y	7	COM	Died
10	M	III	48	130	N	5	OME	Alive
11	F	II	54	78	N	4	OME	Alive

RT dose: radiotherapy dose (Gy); F: female; M: male; Y: yes; N: no; latency: the latency period between the final RT session and the occurrence of BPPV; COM: chronic otitis media; OME: otitis media with effusion.

**Table 2:** Comparison of clinical characteristics between patients with and without BPPV.

Characteristics	With BPPV	Without BPPV	*P* value
Age (years)*	51 (32–65)	50 (34–62)	
Gender (M/F)	7/4	7/4	
RT dose			
Once 70–78 Gy	4 (36.4%)	10 (90.9%)	0.027
Twice 120–130 Gy	7 (63.6%)	1 (9.1%)	
Neck fibrosis	3 (27.3%)	4 (36.4%)	1.000
Otitis media	COM: 4 (36.4%)	COM: 1 (9.1%)	0.011
	OME: 5 (45.5%)	OME: 1 (9.1%)	
SNHL	8 (72.7%)	1 (9.1%)	0.009

*Data presented with median and range; SNHL: sensorineural hearing loss; COM: chronic otitis media; OME: otitis media with effusion.

**Table 3:** Clinical manifestations of 11 irradiated NPC patients with BPPV.

Patient	BPPV type	Diagnostic maneuvers	Provoked nystagmus	Therapeutic procedure	Outcome
1	PC(L)	Dix-Hallpike test	Torsional upbeating	CRP	Resolved
2	HC(R)	Supine roll test	Geotropic horizontal	GM	Resolved
3	PC(R)	Dix-Hallpike test	Torsional upbeating	CRP	Resolved
4	PC(R)	Dix-Hallpike test	Torsional upbeating	SM	Resolved
5	PC(R)	Dix-Hallpike test	Torsional upbeating	CRP	Resolved
6	HC(L)	Supine roll test	Apogeotropic horizontal	GM	Resolved
7	PC(R)	Dix-Hallpike test	Torsional upbeating	CRP	Resolved
8	PC(L)	Dix-Hallpike test	Torsional upbeating	SM	Resolved
9	PC(R)	Dix-Hallpike test	Torsional upbeating	CRP	Resolved
10	PC(L)	Dix-Hallpike test	Torsional upbeating	CRP	Resolved
11	HC(L)	Supine roll test	Geotropic horizontal	LM	Resolved

PC: posterior canal; HCs: horizontal canal; L: left; R: right; CRP: canalith repositioning procedure (Epleys maneuver); SM: Semont's maneuver; LM: Lemperts maneuver; GM: Gufoni maneuver.
